# Expectancy effects on serotonin and dopamine transporters during SSRI treatment of social anxiety disorder: a randomized clinical trial

**DOI:** 10.1038/s41398-021-01682-3

**Published:** 2021-11-03

**Authors:** Olof R. Hjorth, Andreas Frick, Malin Gingnell, Johanna M. Hoppe, Vanda Faria, Sara Hultberg, Iman Alaie, Kristoffer N. T. Månsson, Jörgen Rosén, Margareta Reis, Kurt Wahlstedt, My Jonasson, Mark Lubberink, Gunnar Antoni, Mats Fredrikson, Tomas Furmark

**Affiliations:** 1 Department of Psychology, Uppsala University, Uppsala, Sweden; 2 The Beijer Laboratory, Department of Neuroscience, Psychiatry, Uppsala University, Uppsala, Sweden; 3 Department of Neuroscience, Psychiatry, Uppsala University, Uppsala, Sweden; 4 Center for Pain and the Brain, Department of Anesthesiology Perioperative and Pain Medicine, Boston Children’s Hospital, Harvard Medical School, Boston, MA USA; 5 Smell & Taste Clinic, Department of Otorhinolaryngology, TU Dresden, Dresden, Germany; 6 Department of Neuroscience, Child and Adolescent Psychiatry, Uppsala University, Uppsala, Sweden; 7 Center for Psychiatry Research, Department of Clinical Neuroscience, Karolinska Institutet, Stockholm, Sweden; 8 Center for Lifespan Psychology, Max Planck Institute for Human Development, Berlin, Germany; 9 Max Planck UCL Center for Computational Psychiatry and Ageing Research, Berlin/London, UK; 10 Department of Biomedical And Clinical Sciences, Linköping University, Linköping, Sweden; 11 Department of Clinical Chemistry and Pharmacology, Skåne University hospital, Lund, Sweden; 12 Department of of Surgical Sciences/Nuclear Medicine and PET, Uppsala University, Uppsala, Sweden; 13 Department of Medicinal Chemistry, Uppsala University, Uppsala, Sweden

**Keywords:** Predictive markers, Human behaviour, Molecular neuroscience

## Abstract

It has been extensively debated whether selective serotonin reuptake inhibitors (SSRIs) are more efficacious than placebo in affective disorders, and it is not fully understood how SSRIs exert their beneficial effects. Along with serotonin transporter blockade, altered dopamine signaling and psychological factors may contribute. In this randomized clinical trial of participants with social anxiety disorder (SAD) we investigated how manipulation of verbally-induced expectancies, vital for placebo response, affect brain monoamine transporters and symptom improvement during SSRI treatment. Twenty-seven participants with SAD (17 men, 10 women), were randomized, to 9 weeks of overt or covert treatment with escitalopram 20 mg. The overt group received correct treatment information whereas the covert group was treated deceptively with escitalopram, described as an active placebo in a cover story. Before and after treatment, patients underwent positron emission tomography (PET) assessments with the [^11^C]DASB and [^11^C]PE2I radiotracers, probing brain serotonin (SERT) and dopamine (DAT) transporters. SAD symptoms were measured by the Liebowitz Social Anxiety Scale. Overt was superior to covert SSRI treatment, resulting in almost a fourfold higher rate of responders. PET results showed that SERT occupancy after treatment was unrelated to anxiety reduction and equally high in both groups. In contrast, DAT binding decreased in the right putamen, pallidum, and the left thalamus with overt SSRI treatment, and increased with covert treatment, resulting in significant group differences. DAT binding potential changes in these regions correlated negatively with symptom improvement. Findings support that the anxiolytic effects of SSRIs involve psychological factors contingent on dopaminergic neurotransmission while serotonin transporter blockade alone is insufficient for clinical response.

## Introduction

Selective serotonin reuptake inhibitors (SSRIs) are commonly prescribed for depression and anxiety but it has been widely debated to what extent SSRI efficacy can be attributed to expectancies of improvement—a key mechanism of placebo effects [1–6]. This question has been discussed extensively in the field of depression, but it is relevant also for anxiety conditions [7, 8] including social anxiety disorder (SAD) [9]. Meta-analyses support that SSRIs are efficacious for these disorders [10, 11] but the clinical effect of SSRIs in double-blind RCTs may, at least partly, reflect an enhanced placebo response because of perceived side effects by participants in the active drug arm, compromising the integrity of the blind and increasing response expectancies [12]. While this notion has been questioned [6, 13], it is supported by trials using active placebo, mimicking the side effects of the active substance, and by experimental research demonstrating that expectancies affect therapeutic outcomes [14–16]. Further research is needed to clarify the magnitude of the SSRI clinicial effect, to what extent it can be attributed to the drug itself, and the neural mechanisms underlying symptom remission with SSRIs.

Research designs involving deception have been used to separate drug from expectancy effects in clinical as well as neuroimaging trials [9, 15–18]. We previously demonstrated enhanced anti-anxiety effects of overt as compared to covert SSRI treatment with escitalopram in patients with SAD [9]. Patients were treated with equivalent clinical doses of escitalopram for 9 weeks, but only one group was correctly informed about the treatment received and its effectiveness. Using a credible cover story, the other group was led to believe that they were treated with an “active placebo” (a neurokinin-1 receptor antagonist) expected to induce similar side effects as the SSRI while lacking anxiolytic properties. In the present study, we further investigated the therapeutic mechanisms underlying SSRI efficacy by analyzing how serotonin and dopamine transporters (DATs) are affected by response expectancies.

The SSRIs are held to exert their therapeutic effects by blocking serotonin uptake via the serotonin transporter (SERT) [19] and clinical doses typically result in 76–85% SERT occupancy in the striatum [20]. However, the downstream therapeutic mechanisms of action are still not elucidated, and in this respect dopaminergic mechanisms may also be important as the serotonin and dopamine systems have reciprocal functional influences on each other [21, 22]. SAD patients show increased expression and co-expression of SERTs and DATs in comparison to healthy controls [23]. Molecular neuroimaging studies suggest that SSRIs exert effects also on the DAT [24–28]. It is, however, unclear to what extent SERTs and DATs are affected by the SSRI itself or by psychological processes like expectancies.

Here, in a subsample of our previous SSRI deception study of SAD [9], we examined if giving correct or incorrect information about the drug affects serotonergic and dopaminergic neurotransmission as assessed with positron emission tomography (PET) and the highly selective radioligands [^11^C]DASB and [^11^C]PE2I, probing SERTs and DATs respectively. Effects on monoamine transporter proteins and clinical responsiveness were evaluated when escitalopram was administered with and without clear expectations of improvement, i.e., overt vs. covert SSRI treatment.

## Methods

### Participants

We studied a PET subsample of a previously reported SAD treatment cohort, and for methodological details, we refer to that paper [9]. Also, PET baseline comparisons of SAD patients vs. healthy controls have been reported elsewhere [23]. Here, 27 right-handed patients with SAD (17 men, 10 women; mean ± SD age, 31.1 ± 10.3 years) underwent [^11^C]DASB and [^11^C]PE2I PET imaging before and after 9 weeks of escitalopram treatment—see Fig. S1 and Table S1 in the Supplementary. Of these, one female participant could not be included in posttreatment [^11^C]DASB analyses due to scanner failure. In addition to the included sample, two patients were assessed by PET at baseline but were excluded from analyses due to magnetic resonance imaging (MRI) contraindications, and withdrawal from the study before completed MRI, respectively. Between March 17th 2014 and May 22nd 2015, participants were recruited through advertisements in newspapers, public billboards and the internet. Exclusion criteria were age <18 or >65 years, earlier PET-scan, contraindications for MRI, pregnancy, menopause, substance abuse or dependency, any ongoing severe somatic disease or serious psychiatric disorder, and ongoing or recently terminated (<3 months) psychiatric treatment.

Participants were screened using an extensive online form and those not meeting the initial exclusion criteria were administered an excerpt from the Structured Clinical Diagnostic Interview for the DSM-IV [29] and the full Mini-International Neuropsychiatric Interview [30] via telephone to verify a DSM-IV primary diagnosis of SAD. Social anxiety symptom severity was measured with the self-report version of the Liebowitz Social Anxiety Scale [31], LSAS-SR (pre-treatment mean ± SD: 84.96 ± 20.37).

### Treatment design

**Fig. 1:**
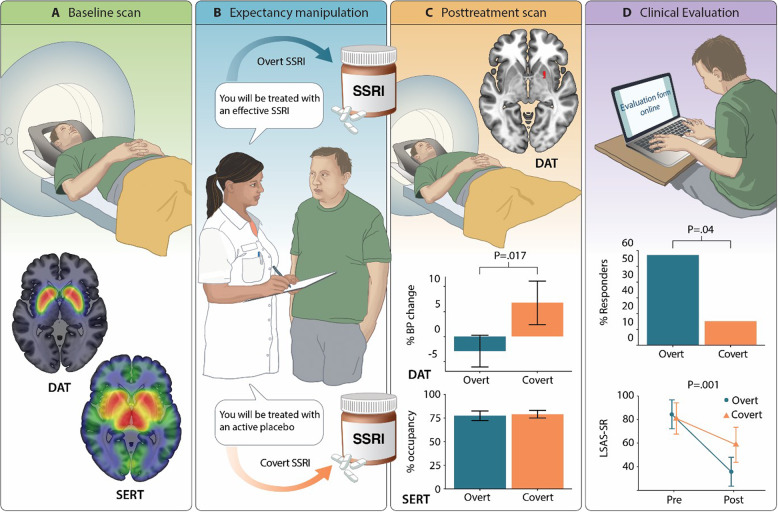
Study design and main results. **A** Shows the whole-sample distribution of serotonin (SERT) and dopamine (DAT) transporters, expressed as non-displacable binding potentials (BP_ND_) at the baseline PET assessment. **B** Illustrates the experimental manipulation; high or low response expectancies were induced by different verbal instructions. All patients were then treated under randomized conditions with escitalopram 20 mg for 9 weeks, correctly described as an effective SSRI for the overt group and incorrectly described as an active placebo in the covert group. **C** Shows the treatment effects on PET measures. Overt as compared to covert SSRI treatment resulted in lowered DAT availability, the significant cluster in the right putamen/pallidum is shown together with percent BP_ND_ change from pre- to posttreatment. In contrast, the average escitalopram SERT occupancy levels were similar in both groups after treatment. **D** Shows the results of the clinical evaluation. Overt as compared to covert treatment resulted in a significantly higher percentage responders and lowered (pre-post) social anxiety as assessed with the Liebowitz Social Anxiety Scale, self-report (LSAS-SR) administered online. Error bars reflect 95% confidence intervals.

The study was an investigator-initiated clinical trial with SAD patients, matched for age and sex, randomized to either overt (*n* = 14) or covert (*n* = 13) SSRI-treatment. The experimental manipulation was verbal instructions of whether the drug was expected to be effective or not. After baseline scans (Fig. 1A), one group was instructed that they would receive escitalopram, demonstrated to be effective for SAD, and the other group that they would receive a non-effective neurokinin-1-receptor antagonist, in the cover story described as an active placebo with similar side effects as escitalopram but out of which no symptom-improvement could be expected (Fig. 1B) [9]. However, both groups were treated with 20 mg escitalopram per day, starting with 10 mg the first week. All accepted their allocated group. All participants and observers were blinded to manipulation except the study clinician who supervised medication and debriefed participants when the cover story was revealed [9].

Participants revisited the clinic after 1 week and were then handed their supply of the medication for the remainder of the study period. Blood serum analyses were performed to examine escitalopram and metabolite concentrations at posttreatment and compliance was further assessed by counting of remaining capsules at the posttreatment visit—see [14]. Treatment randomization and preparation of escitalopram was prepared by APL, Stockholm, Sweden. The study was approved by the Regional Ethical Review Board in Uppsala, the Radiation Safety Committee at Uppsala University Hospital and the Medical Products Agency in Sweden. All participants were informed both verbally and in writing regarding study objectives, comparing escitalopram and “active placebo”, as well as side-effects of drugs and risks of neuroimaging methods. The full written and verbal information is provided in the supplementary appendix to our previous paper [9]. All participants were offered additional treatment with internet-delivered cognitive-behavior therapy after the study period. Written consent was required for inclusion.

### Imaging procedure

#### Positron emission tomography

A Siemens ECAT EXACT HR + (Siemens/CTI) was used to acquire the PET images with 63 contiguous planes of data and slice thickness of 2.46 mm resulting in a total axial field of view of 155 mm. Participants fasted for at least 3 h and refrained from alcohol, nicotine and caffeine for at least 12 h before the scan. At posttreatment, participants were instructed to take the escitalopram dose 24 h before the PET scan. Participants were positioned supine in the scanner with their head gently fixated and a venous catheter for tracer injections was inserted. A 10 min transmission scan for attenuation correction was performed using three retractable germanium (^68^Ge) rotating line sources.

Participants were injected with on average 327 ± 27 MBq of [^11^C]PE2I (N-(3-iodopro-2E-enyl)-2b-carbomethoxy-3b-(4-methyl-phenyl)nortropane) through an intravenous bolus and 22 frames of data were acquired over 80 min of data (4 × 60 s, 2 × 120 s, 4 × 180 s, 12 × 300 s). Following a 45–60 min waiting period to allow for sufficient decay of the radioactivity (i.e., >6 radioactive half-lifes), acquisition commenced for [^11^C]DASB (3-amino-4-(2-dimethylaminomethylphenylsulfanyl)-benzonitrile), using an identical injection procedure and an average activity of 333 ± 20 MBq. In total, 22 frames of data were acquired over 60 min (1 × 60 s, 4 × 30 s, 3 × 60 s, 4 × 120 s, 2 × 180 s, 8 × 300 s).

#### Magnetic resonance imaging

Participants underwent an anatomical T1-weighted MR scan used for anatomical referencing of PET data (echo time (TE) = 50 ms; repetition time (TR) = 500 ms; Field of view = 240 × 240 mm^2^; voxel size = 0.8 × 1.0 × 2.0 mm^3^; 170 contiguous slices) on a Philips Achieva 3.0 T whole body MR-scanner (Philips Medical Systems, Best, The Netherlands) with an 8-channel head-coil. Five participants were scanned with a 32-channel head-coil due to a scanner upgrade.

### PET data preprocessing

Ordered subset expectation maximization with six iterations and eight subsets and a 4 mm Hanning post-filter with appropriate corrections was used to reconstruct dynamic images. Voxel-wise parametric images of non-displaceable binding potentials (BP_ND_) were calculated for both radioligands with the cerebellum as reference region using reference Logan [32] for [^11^C]DASB (time interval 30–60 min) and receptor parametric mapping [33] for [^11^C]PE2I. Cerebellar gray matter was selected as reference region for both radioligands because of the negligible levels of SERTs and DATs. It was automatically outlined on each participant’s anatomical T1-weighted image using the PVElab software [34].

The pre and post treatment [^11^C]DASB BP_ND_ and [^11^C]PE2I BP images were co-registered to the anatomical T1-weighted MR image using Statistical Parametric Mapping 8 (SPM8; (Wellcome Department of Cognitive Neurology, University College London, www.fil.ion.ucl.ac.uk) implemented in Matlab (Mathworks Inc., Nantucket, MA, USA). The T1-image was then segmented and normalized to the Montreal Neurological Institute (MNI) standard space and the transformation parameters applied to the [^11^C]DASB and [^11^C]PE2I BP_ND_ images, resulting in parametric images with 2 mm isotropic voxels. Images were then smoothed using a 12 mm Gaussian kernel.

### Statistical analysis

Behavioral treatment outcome was assessed with mixed repeated measures ANOVA of LSAS-SR, and Fisher’s exact test of number of responders fulfilling the criteria for clinically significant improvement [35]. Participants were deemed to be responders if they were within two standard deviations of the normal population after treatment (LSAS score < 39), and exhibited a Reliable Change Index larger than 1.96 [35].

As in our recent PET-study [23], the a priori regions of interest (ROIs) for both radiotracers were the amygdala, hippocampus, caudate nucleus, putamen, nucleus accumbens (NAcc), pallidum and thalamus, and for [^11^C]DASB also the anterior cingulate cortex (ACC), insula cortex and raphe nuclei. Anatomical regions were defined using the Automated Anatomical Labeling library from the Wake Forest University Pickatlas [36] except for the NAcc and raphe nuclei which were defined by the Hammersmith atlas [37] and PVElab software [34] respectively.

For voxel-wise analyses, SERT occupancy [(pre-post)/pre] images and percentage change in DAT binding potential [(post-pre)/pre] images were calculated. To examine group differences before treatment and changes with treatment, two-sample *t*-tests were performed on BP_ND_ data for both tracers separately in SPM8 with age and sex as covariates. Correlations between LSAS-SR and brain measures were performed using Pearson’s product-moment correlations for [^11^C]DASB and [^11^C]PE2I separately. The statistical threshold was set at *P* < 0.05 and analyses were corrected for familywise error (FWE) within the ROIs.

We used Fisher-transformed partial Pearson’s product-moment correlations to examine voxel-wise relations between SERT occupancy and percent change in DAT BP_ND_ with the statistical threshold set to *P* < 0.05 [23]. Analyses were performed in MatlabR2018a.

## Results

### Serotonin transporter binding

Distribution of [^11^C]DASB binding, probing SERT availability at baseline, is shown in Fig. 1A. Groups did not differ in initial SERT BP_ND_. After expectancy manipulation and 9 weeks of treatment (Fig. 1B), no between-group (overt vs. covert) differences in SERT occupancy were detected and escitalopram SERT occupancy was significant in all evaluated ROIs with an average of 78% when accounting for total volume (Fig. 1C, Table S2). There were no correlations (*P* > 0.10) between SERT occupancy and symptom improvement as assessed with LSAS-SR.

### Dopamine transporter binding

**Fig. 2:**
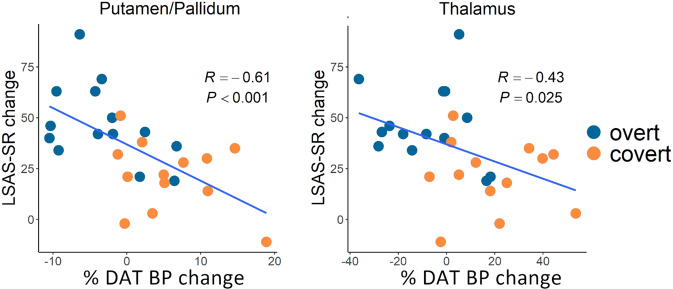
Brain-behavior correlations. Significant correlations are shown between decreased dopamine transporter (DAT) availability, expressed as percent pre-post change in binding potential (BP), and symptom improvement expressed as higher scores on the Liebowitz Social Anxiety Scale self-report (LSAS-SR) before as compared to after treatment. Significant correlations were noted in the right putamen/pallidum cluster (left panel) and in the left thalamus (right panel).

Distribution of [^11^C]PE2I binding, probing DAT availability at baseline, is shown in Fig. 1A. Groups did not differ in initial DAT BP_ND_ in any region except for the right thalamus (MNI x,y,z: 4,−10,10, *P*_FWE_ = 0.001, *Z* = 4.13, 1584 mm^3^). Following expectancy manipulation and treatment (Fig. 1B), between-group analyses showed a differential response with relative decreases in DAT BP_ND_ in the overt group and increases in the covert group, in the right putamen, extending into pallidum, and also in the left thalamus (Fig. 1C, Table 1). Follow-up analysis showed that reduced DAT binding in these clusters correlated with social anxiety symptom improvement (Fig. 2). In addition, within-group analyses revealed significantly decreased (pre-post) DAT BP_ND_ in the right amygdala in the overt SSRI group and increased DAT BP_ND_ in the bilateral pallidum, left thalamus and left hippocampus in the covert group (Table 1). Between-group differences in the right amygdala (MNI x,y,z: 24,2,−12, *P* = 0.017, *Z* = 2.11, 280 mm^3^), and left hippocampus (MNI x,y,z: 18,−30,−4, *P* = 0.009, *Z* = 2.34, 32 mm^3^) were significant at an uncorrected statistical threshold, with relatively higher increases in binding in the covert group.

**Table 1:** Brain regions showing differences in dopamine transporter binding potential change after overt and covert SSRI treatment.

	Hemisphere	MNI x, y, z			*Z*	*P*	Cluster volume
*Within groups*							
*Overt Pre* *>* *Post*							
Amygdala	Right	34	4	−20	2.94	0.035	8
*Covert Pre* *<* *Post*							
Hippocampus	Left	−22	−36	4	3.27	0.046	8
Pallidum	Left	−22	−2	−4	2.90	0.041	24
Pallidum	Right	24	2	−4	2.80	0.050	8
Thalamus	Left	−20	−30	4	3.80	0.006	240
*Between groups*							
*Covert* *>* *Overt*							
Putamen	Right	22	8	−4	3.46	0.017	144
Pallidum	Right	22	4	−2	3.10	0.020	72
Thalamus	Left	−20	−30	4	3.40	0.018	56

*MNI* Montreal Neurological Institute coordinate; Cluster volume in mm^3^

### Concomitant changes in serotonin-dopamine transporter binding with treatment

**Fig. 3:**
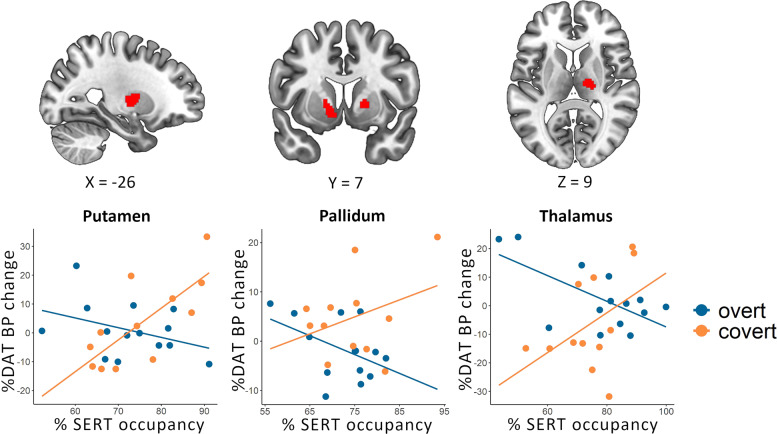
Concomitant serotonin-dopamine transporter changes with overt and covert SSRI treatment. Significant group differences in correlations between percent sertonin transporter (SERT) occupancy and change in dopamine transporter binding (DAT BP) from pre-to post-treatment were noted in the left putamen, bilateral pallidum, and right thalamus (top panel). Scatterplots are shown in the lower panel.

Correlations between SERT occupancy and percentage change in DAT BP_ND_ within each treatment group, as well as significant group differences in these correlations, are listed in Table 2. Significant group differences were noted in the bilateral pallidum, left putamen and right thalamus (Fig. 3). Level of SERT occupancy correlated with decreased DAT BP_ND_ in the overt group and increased DAT BP_ND_ in the covert SSRI group. To statistically evaluate involvement of SERT-DAT interactions in response expectancies, follow-up logistic regression analyses were conducted with transporter changes as independent variables and group (overt/covert) as dependent variable. These confirmed that inclusion of SERT×DAT interaction terms to the models with main effects of SERT and DAT, drastically increased the McFadden *R*^2^ explained variance in all regions (interaction/main effects: putamen = 0.28/0.02; left pallidum = 0.19/0.03, right thalamus = 0.41/0.11), except the right pallidum (0.27/0.27).

**Table 2:** Brain regions showing significant correlations between serotonin transporter occupancy and percent dopamine transporter binding potential change after overt and covert SSRI treatment.

	Hemisphere	MNI x, y, z			Covert *r*^a^	Overt *r*^a^	diff *r*^b^	Cluster volume^c^
*Within groups*								
*Overt*								
Putamen	Right	30	4	6		−0.80		9152
Thalamus	Right	20	−18	10		−0.77		1920
Thalamus	Right	4	−8	4		−0.64		384
*Covert*								
Putamen	Left	−24	−8	8	0.84			15808
Thalamus	Right	18	−16	10	0.74			640
Thalamus	Right	10	−8	−2	0.68			576
Thalamus	Left	−18	−22	10	−0.81			640
*Between groups*								
*Covert* *>* *Overt*								
Putamen	Left	−26	−6	8	0.82	−0.53	1.349	6784
Pallidum	Left	−10	6	−4	0.77	−0.46	1.233	3072
Pallidum	Right	18	8	2	0.41	−0.67	1.077	704
Thalamus	Right	20	−18	10	0.65	−0.77	1.417	5760

All analyses are at *p* < 0.05 with age and sex as covariates.

*MNI* Montreal Neurological Institute.

^a^Partial Pearson’s product-moment correlation coefficient *r*.

^b^Differences in Pearson’s *r* correlation coefficient between groups.

^c^Cluster volume in mm^3^.

### Blood serum analyses

Groups did not differ significantly in blood serum concentrations of escitalopram (*t* = −0.78, *P* = 0.44, 95% CI: −60.3–27.2) or S-desmethylcitalopram (*t* = 0.55, *P* = 0.59, 95% CI: −10.1–17.5) at posttreatment—see Supplementary Appendix.

### Clinical evaluation

After treatment, there were significantly more responders in the overt (8/14; 57%) than the covert (2/13; 15%) group (Fisher’s exact test: OR = *0.15, P* = 0.046), according to conservative response criteria [35]—see Fig. 1D. On the main outcome measure (LSAS-SR), groups did not differ in pre-treatment scores (*t*_*(24.71)*_ = 0.44, *P* = 0.67, 95% CI: −12.96–19.94) and ANOVA revealed a significant Group × Time interaction (*F*_*(1,25)*_ = 13.20, 95% CI of group difference = 11.15–40.07, *P* = 0.001) with larger improvement in the overt (M_diff_ ± SD = 47.07 ± 19.23, Cohen’s *d* = 2.33) as compared to the covert (M_diff_ = 21.46 ± 17.25, Cohen’s *d* = 0.93) group over 9 weeks of treatment (Fig. 1D). Thus, as in the larger cohort [9], superiority of overt (>covert) SSRI administration was noted.

## Discussion

Verbally-induced response expectancies had a significant influence on SSRI-efficacy and dopamine, but not serotonin, transporter availability. Overt was clinically superior to covert SSRI-treatment, with almost a fourfold higher response rate, resulting in relatively lowered DAT binding in striatal and thalamic brain regions that correlated significantly with symptom improvement. In contrast, groups did not differ in levels of SERT occupancy after treatment, and escitalopram/S-desmethylcitalopram serum concentrations were also similar.

The present findings support that dopamine neurotransmission is crucially involved in the therapeutic mechanisms of SSRIs and that the anxiolytic properties can be attributed largely to psychological factors. DAT binding in the putamen, pallidum, and thalamus increased with covert SSRI treatment while it decreased in the overt group, with reductions linearly coupled to symptom improvement, suggesting slower clearance and/or increased release of dopamine when expectancies are higher, resulting in better improvement. Previous SPECT studies, including a study of SAD, have generally noted increased striatal DAT binding after acute or stable SSRI treatment [24–28]. It should be noted, however, that the radioligands used in these SPECT studies are affected by SSRIs, and are not as specific and sensitive as the current [^11^C]PE2I PET ligand [38]. Moreover, expectancies were not assessed in previous studies. Other lines of evidence also support that SSRIs have measurable dopaminergic effects, although the direction has varied. For example, in a study of dopaminergic challenges in SAD, an acute dose of pramipexole but not sulpiride, attenuated anxiety levels during a behavioral test in SSRI-treated patients, suggesting desensitization of dopamine D3 receptors [39]. Some side effects of SSRIs have previously been indentifed as dopamine-dependent [40]. Further, animal studies show that serotonin agonists and SSRIs increase extracellular dopamine levels in the striatum, hypothalamus and prefrontal cortex [41, 42] and that SSRI antidepressant effects are abolished by dopamine depletion [43]. Previous research also indicates promiscuity between monoamine transporters [22] and that serotonin can be transported by DATs when SERTs are blocked by SSRIs [44]. This may be counterbalanced by decreased DAT availability when response expectancies are high, or reinforced when expectancies are low.

Striatal regions are important for reward processing, receiving input from the thalamus while also relaying information to the thalamus through pallidum [45]. Higher expectancies with overt treatment may come with more optimistic cognitions, remoralization, enhanced approach motivation and willingness to engage in self-exposure, enhancing reinforcement learning and dopamine-dependent reward function. Indeed, reward expectancy and approach motivation activate the striatal dopamine system [46, 47] as do placebo effects [47]. Conversely, animal studies report reduced striatal dopamine release during passive coping with stressful situations [48]. The overt group also exhibited significantly decreased DAT BP_ND_ in the right amygdala, a central hub in threat processing. The association between decreased amygdala-striatal DAT availability and better anxiolytic effects is congruent with our recent PET study in which baseline DAT BP_ND_ correlated positively with anxiety severity, indicative of dopamine hypoactivity in SAD [23]. Dopaminergic hypofunction has also been suggested to underlie at least some subgroups of treatment resistant depression for which dopamine agonists could be effective [49].

The present findings suggest that pharmacologic SERT-blockade is, by itself, not sufficient for adequate clinical improvement. Because the SSRIs are effective in SAD [10] and block the SERT in a dose-dependent manner [20], and because PET studies show increased SERT availability in SAD [23, 50], it could be expected that the anti-anxiety effects of SSRIs are SERT-mediated. However, despite the large difference in clinical efficacy, SERT occupancy was equally high in the overt and covert SSRI groups in all evaluated brain regions, and did not correlate with reduced social anxiety. This was not explained by attrition or poor SSRI compliance as both groups had comparable and expected blood serum concentrations of escitalopram and S-desmethylcitalopram. Consistently, several molecular imaging studies have failed to demonstrate a relationship between SERT occupancy and clinical response to SSRIs [20, 51, 52]. Similary, pharmacologic SERT blockade occurs within hours after acute SSRI intake while the clinical response is delayed several weeks [53]. Nonetheless, as some improvement occurred also in the covert group [9], ample SERT occupancy could still be a prerequisite for SSRI-induced anxiety relief but other mechanisms are also likely to be involved. A previous study of SSRI-treated patients with SAD reported that lowering of serotonin by tryptophan depletion increased anxiety induced by an autobiographical script, but not by a stressful speaking task [54]. In contrast, PET data from our group suggested that serotonin synthesis was reduced and tied to symptom improvement following SSRI and other pharmacological treatments [55]. Here, we found that superior improvement with overt SSRI administration was tied to decreased DAT availability occurring in parallel to increased SERT occupancy e.g., in the striatum. This suggests that serotonin-DAT interactions are involved, not only in the pathogenesis of SAD [23], but also in response expectancies. The full clinical SSRI response may thus result from expectancy effects on dopamine and serotonin-dopamine interactions, in addition to pharmacological SERT blockade. The drug-expectancy relation could be additive or synergistic [52]. It should be noted that PET-data on transporter proteins are limited to brain regions with adequate tracer uptake and do not provide detailed information about neural signaling, also preventing conclusion about dynamics within and across specific serotonin and dopamine paths as well as tonic-phasic interplay. Further research on pre- and postsynaptic processes is needed to clarify how the monoamines contribute to anxiety and symptom improvement with treatment. The complexity of this issue calls for studies that use a variety of methodologies like multimodal neuroimaging, genetic approaches and pharmacological challenges.

The sample size was relatively small in the present study, due to high costs involved with PET, thereby restricting statistical power. This could increase the risk for type 1 and 2 errors, i.e., either that the between-group null findings on SERT occupancy were false negatives or that significant DAT results were false positives. Since levels of SERT occupancy were highly similar, it is unlikely that significant overt-covert group differences would have emerged with an increased number of subjects. With regard to DAT changes, we observed significant between-group differences as well as significant correlations with symptom improvement at the behavioral level. Moreover, the treatment-related SERT-DAT correlations in striatal and thalamic regions were in opposite direction in the two groups. This coherent pattern of results supports that overt vs. covert SSRI-treatment had dissimilar effects on dopaminergic signaling, arguing against false positives although replication in a larger sample is warranted.

Among the study limitations it should also be mentioned that we, for ethical and practical reasons, could only use two of the four arms in the balanced placebo design [16]. Thus the “told SSRI/given placebo”, and “told placebo/given placebo” conditions were lacking. Also, we did not measure expectancies during the course of treatment because we were wary that this would reveal the study design [9]. Thus, we could not evaluate the relationship between subjective expectancies and imaging or clinical outcomes. Assessment of clinical efficacy was based essentially on a subjective self-report measure (LSAS-SR) and additional objective measures, like cortisol levels or heart rate variability, could have been added. Finally, the generalizability of the present results to other disorders, pharmaceuticals, or treatment modalities is not known and we cannot determine if the SSRI has a long-term or normalizing effect on transporter densities. This would require additional measurements after drug discontinuation.

In conclusion, the anti-anxiety properties of SSRIs appear to be largely dependent on expectancy effects on dopamine signaling while SERT blockade is not sufficient for symptom remission. This provides new insights on the key therapeutic mechanisms of SSRIs, incorporating psychological effects on dopamine neurotransmission.

## Supplementary information

Supplementary material
