# Supplementary material for: Expectancy effects on serotonin and dopamine transporters during SSRI treatment of social anxiety disorder: a randomized clinical trial
Source: Transl Psychiatry. 2021 Nov 3;11:559. doi: 10.1038/s41398-021-01682-3 (PMC8566580; doi:10.1038/s41398-021-01682-3)
Supplement: Supplementary file 1 — Supplementary material [file 41398_2021_1682_MOESM1_ESM.docx]

**Supplementary Appendix**

This appendix has been provided by the authors to give readers additional information about their work.

Supplement to: Hjorth OR, Frick A, Gingnell M, *et al.* Expectancy effects on serotonin and dopamine transporters during SSRI treatment of social anxiety disorder: A randomized clinical trial.

**SUPPLEMENTARY APPENDIX**

| **TABLE OF CONTENT** | **Page** |
| --- | --- |
| **SUPPLEMENTARY RESULTS** | 3 |
| Descriptive characteristics | 3 |
| Serotonin transporter occupancy across regions | 3 |
| Compliance with escitalopram | 3 |
| **Table S1** | 4 |
| **Table S2** | 5 |
| **Figure S1** | 6 |

**SUPPLEMENTARY RESULTS**

**Descriptive characteristics**

Descriptive data for the overt and covert SSRI groups are listed in Table S1, and the CONSORT flow diagram is shown in Figure S1.

**Serotonin transporter occupancy across regions**

Levels of SERT occupancy following treatment with escitalopram are listed in in Table S2.

**Compliance with escitalopram**

At post-treatment, the overt and covert SSRI groups did not differ in blood serum concentrations (nMol/l) of escitalopram (overt M±SD: 84.4±51.3; covert: 101.0±58.3; *t*=-0.78, *P*=.44) or S-desmethylcitalopram (overt M ±SD: 47.1±19.6; covert: 43.5±15.1; *t*=0.55, *P*=.59). There was no correlation (*R*=.13, *P*=.51) or group difference in the correlations (*R* difference = .18, *P*=.54) between escitalopram concentration and symptom improvement. A trend-level positive relationship between S-desmethylcitalopram concentration and symptom improvement was found (*R*=.35, *P*=.07) but groups did not differ (*R* difference = .10, *P*=.78).

**Table S1.** Descriptive statistics.

|  | Overt SSRI | Covert SSRI |
| --- | --- | --- |
| N | 14 | 13 |
| Mean (SD) age | 31.7 (11) | 30.4 (10) |
| Sex, n (%) |  |  |
| Male | 9 (64) | 8 (62) |
| Female | 5 (36) | 5 (39) |
| Other | 0 | 0 |
| Comorbidity, n (%) |  |  |
| Anxiety disorders | 9 (64) | 4 (31) |
| Depression, mild | 4 (19) | 3 (23) |
| Other | 1 (7) | 1 (8) |
| Previous SSRI treatment | 3 (21) | 2 (15) |
| Education, n (%) |  |  |
| At least college degree | 10 (71) | 7 (54) |
|  |  |  |

**Table S2.** Percent intraregional serotonin transporter occupancy [mean (SD)] by escitalopram in the overt and covert treatment groups.

| Region | Overt | Covert | Total^1^ |
| --- | --- | --- | --- |
| Amygdala | 87 (11) | 94 (12) | 90 (12) |
| Hippocampus | 91 (13) | 94 (11) | 92 (13) |
| NAcc | 85 (9) | 87 (8) | 86 (9) |
| Caudate | 94 (15) | 96 (13) | 95 (14) |
| Putamen | 73 (7) | 74 (7) | 73 (7) |
| Pallidum | 71 (8) | 72 (9) | 71 (8) |
| Thalamus | 73 (9) | 75 (6) | 74 (8) |
| Insula | 78 (12) | 80 (9) | 79 (10) |
| ACC | 80 (20) | 79 (15) | 80 (18) |
| Raphe nuclei | 85 (12) | 89 (9) | 87 (11) |

^1^Total occupancy = 78% when accounting for total volume; NAcc = Nucleus Accumbens; ACC = Anterior Cingulate Cortex


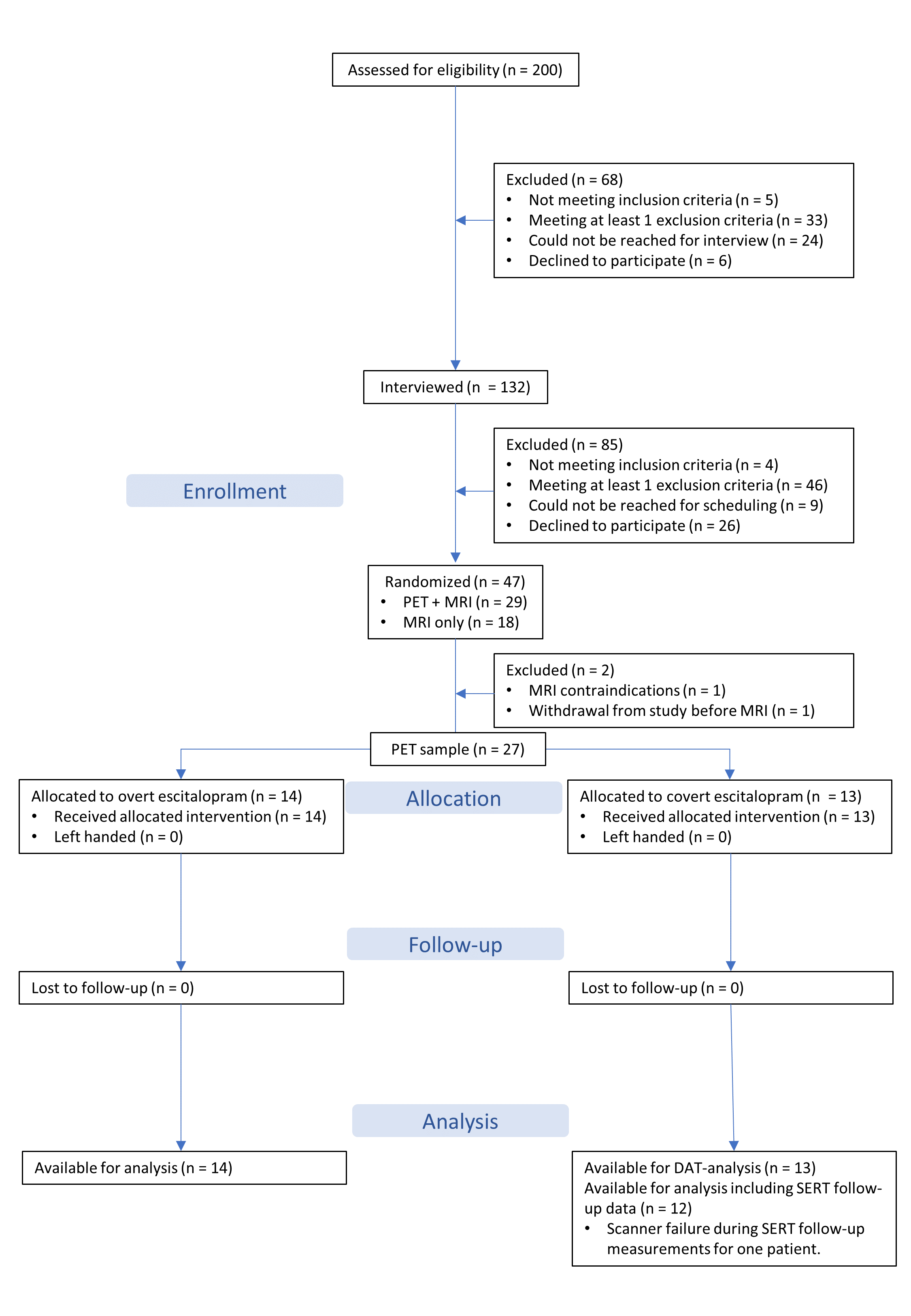


**Figure S1**. CONSORT flow diagram.
